# A Co-Operative Store in New York

**Published:** 1876-07

**Authors:** Charles Barnard


### *A CO-OPERATIVE STORE IN NEW YORK.*

A co-operative association, now in successful operation in New York City, exhibits some features of interest in showing another method. In November, 1875, thirty gentlemen of means and position united under the laws of the State, and opened a co-operative store on Sixth Avenue for their own use and benefit. Each member contributed one hundred dollars in cash, and, under the management of a Board of Directors, a competent manager and four assistants were engaged at reasonable wages. A small store was hired, a choice stock of groceries purchased, a few simple rules prepared, and the store went into operation. By these rules each member makes all his purchases at the store, and either pays cash, or opens an account that must be paid on the first day of each month. The member has nothing to do beyond this. He pays in his hundred dollars, forgoes all interest in it, and expects no bonus, or dividend of any kind. The profit comes in the reduced price of the goods. Once each month the business of the store is examined by an Advisory Board, and if there is a profit over the expenses, the prices are lowered sufficiently to extinguish it. If there is a loss, the prices are raised snfficiently to cover it during the next month. The experiment has, so far, worked smoothly and proved a success. The store not only supplies the members with the best goods, but delivers them free at their residences at a very material reduction from the retail market rates. The store itself is perfectly plain, and is exceptionally neat and attractive. There is no gilding or display, not even a sign, except a card on the door. It is only open by daylight, and is only visited by the members. No member is liable beyond the hundred dollars on joining the association, and any one may withdraw at any time by giving sufficient notice, and may then recover his money, in the form of a gradual abatement on his monthly purchases.—*Charles Barnard, Scribner for June.*

